# Management of Chronic Inflammatory Gingival Enlargement: A Short Review and Case Report

**DOI:** 10.7759/cureus.46770

**Published:** 2023-10-09

**Authors:** Avreet Sandhu, Divya Jyoti, Ritu Malhotra, Tanvi Phull, Haridarshan S Sidhu, Shanteri Nayak

**Affiliations:** 1 Department of Orthodontics and Dentofacial Orthopaedics, Luxmi Bai Institute of Dental Science and Hospital, Patiala, IND; 2 Department of Oral Health Sciences, Postgraduate Institute of Medical Education and Research, Chandigarh, IND; 3 Department of Prosthodontics, ITS Centre for Dental Studies and Research, Ghaziabad, IND; 4 Department of Oral and Maxillofacial Surgery, Gian Sagar Dental College, Patiala, IND; 5 Department of Pediatric Dentistry, Government Dental College, Patiala, IND; 6 Department of Periodontology, Punjab Government Dental College and Hospital, Amritsar, IND

**Keywords:** patient education, plaque induced gingival enlargement, periodontology, external bevel gingivectomy, chronic gingival inflammation, inflammatory gingival enlargement

## Abstract

Inflammatory gingival enlargement, sometimes referred to as gingival hyperplasia or gingival hypertrophy, is an abnormal proliferation of gingival tissues caused by underlying inflammation. It might also be related to long-term periodontitis. Herein, we discuss the case of a young, otherwise healthy male patient wherein the anterior regions of both the upper and lower arches were affected by long-standing gingival growth. The overgrowth was removed, and an excellent aesthetic outcome was achieved, using a surgical procedure termed gingivectomy. After a 15-day follow-up period, the healing process was satisfactory and no negative effects were found.

## Introduction

Due to constant internal and external stimuli, the oral mucosa may undergo a range of illnesses, from reactive to neoplastic [1]. A multifactorial condition called gingival enlargement is brought on by interactions between the environment and the host as well as a number of external factors [2]. These could additionally be the result of trauma, such as calcification, damaged teeth, overhanging restorations, food lodgement, and denture flanges that are overextended [3]. Plaque, systemic hormonal changes, or different blood dyscrasias (such as leukaemia, thrombocytopenia, or thrombocytopathy) can all contribute to its development. Functional problems with speech and mastication as well as psychological and aesthetic problems may be brought on by the degree and extent of the condition [4].

The most common form of enlargement is plaque-induced inflammatory hyperplasia. It can be diffuse or localised, and systemic medications or hormonal changes, like those that occur during puberty or pregnancy, may make it worse [5]. Localised growth can take the form of a sessile or pedunculated mass or nodule that resembles a tumour. Interdental papilla, marginal gingiva, or attached gingiva may all be involved. They might have a spontaneous shrinkage, followed by an aggravation and further growth. Sometimes an uncomfortable ulceration develops in the fold between the mass and the nearby gingiva [6]. Persistent mouth-breathing may potentially result in chronic inflammatory enlargement. It involves the anterior region, primarily the papilla. The mucosa becomes dry as a result of the mouth-breathing habit. There is a distinct difference between gingival involvement and normalcy [6].

Inflammatory enlargement could be acute or chronic. Typically, local deposits build up and cause this. Plaque accumulation-causing factors make an inflammatory enlargement more likely. Interdental papilla and marginal gingival ballooning are the precursors to chronic inflammatory enlargement. Around the affected teeth, an eruption in the shape of a life preserver is seen. This has the potential to grow until it encloses the crown. It is typically painless until the development of trauma or acute illness [6]. This report emphasises the value of gingivectomy as a treatment option for the control of inflammatory gingival enlargement.

## Case presentation

A 23-year-old man presented at the Department of Periodontics at Punjab Government Dental College, Amritsar. The patient's primary complaint at admission was three to four months of gum discomfort, swelling, and bleeding. The patient went to the dentist for the same reason and received astringent gum paint and analgesics a month ago. He brushed his teeth twice a day in a horizontal scrub motion, but for the last two months, the pain and bleeding from the brushing prevented him from doing it effectively. A thorough family history was gathered; however, it was not contributory to the diagnosis. The patient had no underlying conditions that would have accelerated gingival growth. He has never previously been hospitalized or taken medication.

**Figure 1:**
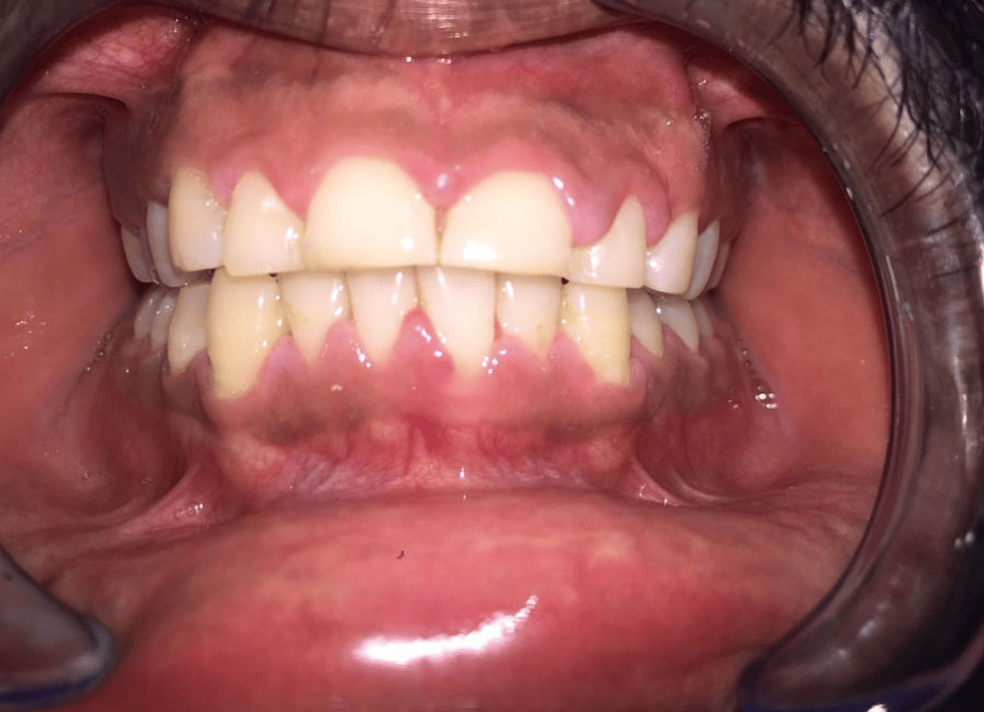
Inflammatory gingival enlargement with respect to 13, 12, 11, 21, 22, 23

**Figure 2:**
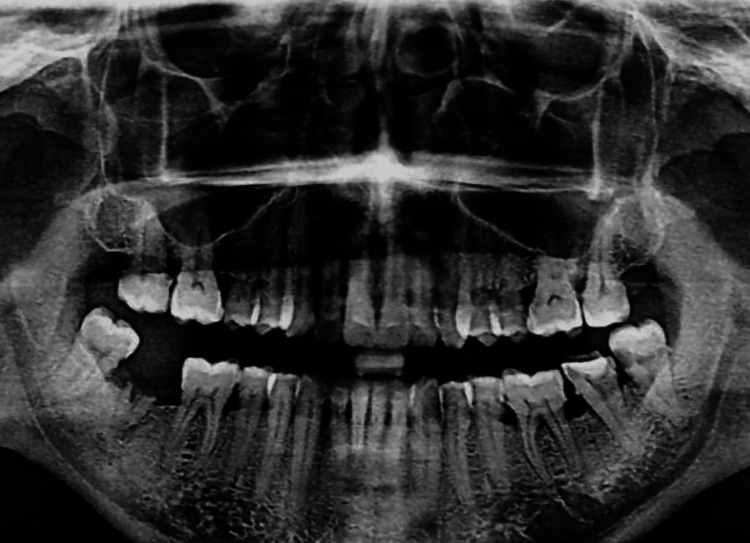
Radiographic image of oral cavity showing no evidence of underlying pathology

Upon clinical examination, it was discovered that the interdental, marginal, and attached gingiva in the maxillary arch was diffusely enlarged from canine to canine (Figure 1). The marginal gingiva had a smooth, lustrous, pinkish-red appearance and was soft and friable. On the slightest provocation, gingival bleeding began. There were no abnormalities found during radiographic and laboratory examinations (Figure 2). A clinical choice was made to start the patient on a good oral hygiene regimen as the initial stage of treatment. Under local anaesthetic, thorough scaling, root planing, and polishing were performed. The patient did not have a mouth-breathing habit. The patient received instructions on proper oral hygiene and how to brush properly with an ultra-soft toothbrush. 

**Figure 3:**
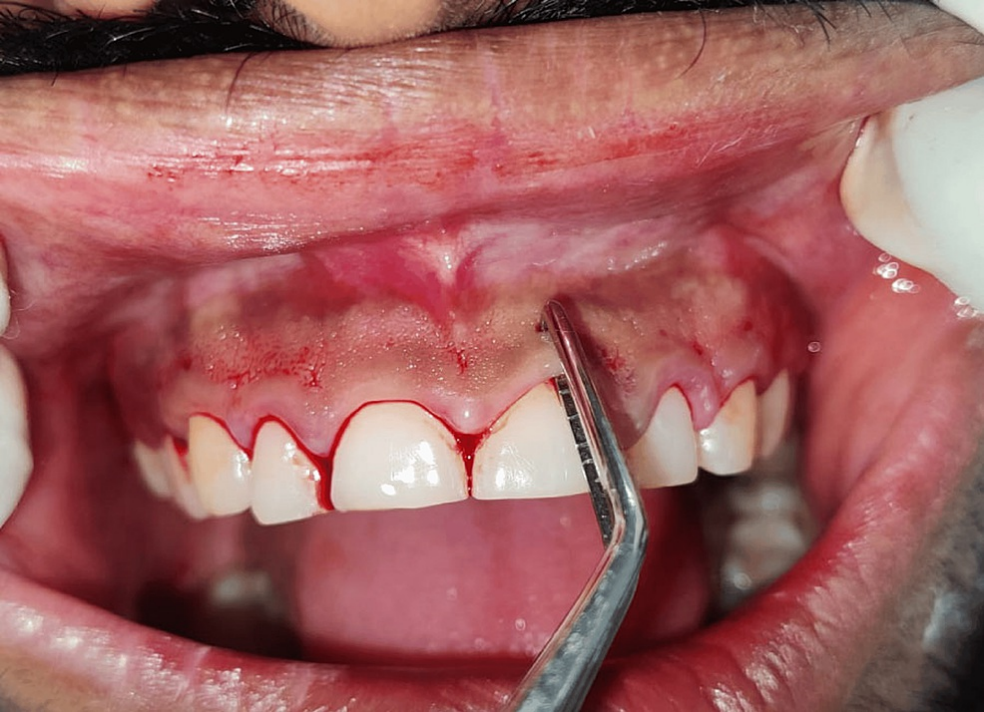
Gingival bleeding points marked using pocket marker

**Figure 4:**
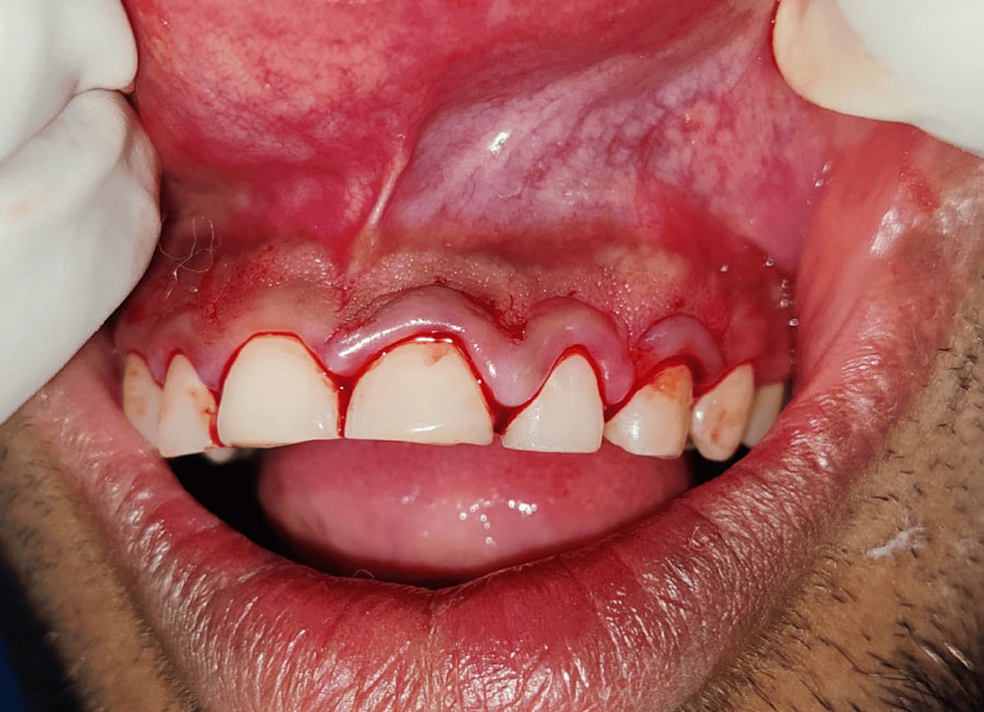
External bevel incision given

**Figure 5:**
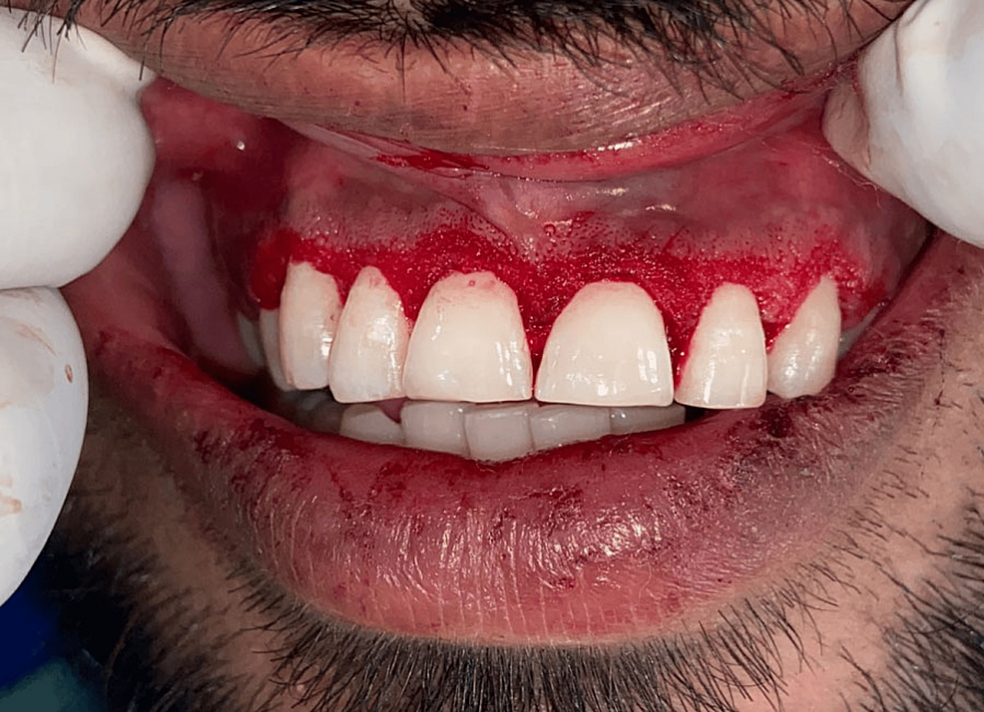
External bevel gingivectomy done

**Figure 6:**
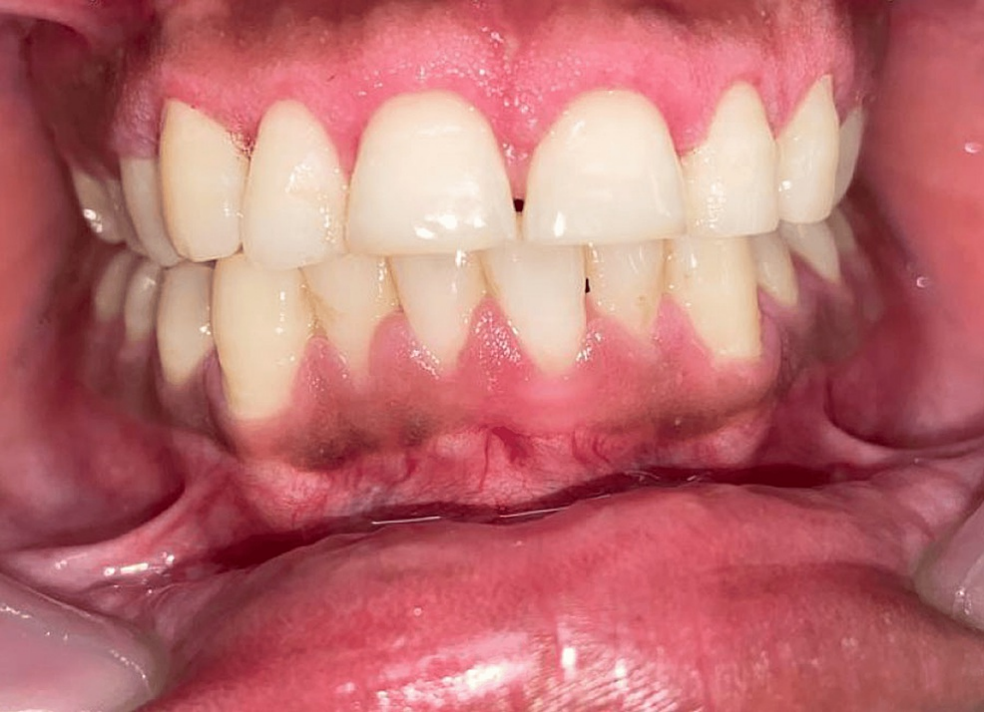
Fifteen days post-operative image

After phase 1 therapy was finished and oral hygiene instructions were reinforced, an external bevel gingivectomy surgery was performed one week later. Local anesthesia was administered before marking any bleeding points with a Crane-Kaplan pocket knife (Figure 3). After locating the bleeding areas, a 45-degree angle was established in the incision by connecting the bleeding spots and making a bevel that started apically and pointed coronally in the direction of the mucogingival junction (Figure 4). Following the external bevel incision (Figure 5), the pocket wall was removed, and a periodontal dressing was placed. There were no suprabony pockets seen during the follow-up visit after two weeks (Figure 6), and the healing went smoothly.

## Discussion

Gingival inflammation can be characterised by a reddish hue of the gingiva that bleeds on probing and a change in the gingiva's firmness from firm to soft. An overgrowth of the gingiva that follows the gingival inflammation resulting from continuous contact with dental plaque is known as inflammatory gingival enlargement which is the most typical type of gingival enlargement [7]. The most common type of chronic inflammatory gingival enlargement is typified by soft, reddish blue gingiva brought on by oedemas and infected cellular infiltration caused by persistent contact with bacterial plaque. This issue can be handled with a periodontal therapy known as scaling and root planing. If the fibrotic components of the chronic inflammatory gingival enlargement do not lessen after phase I therapy, surgical removal of the extra tissue should be taken into consideration [8].

In terms of clinical signs, plaque-induced gingival enlargement frequently appears as bigger gingival contours brought on by oedema, colour changes to red and/or bluish-red, bleeding following probing, and an increase in gingival exudates [9]. Classification of gingival enlargement has four grades, namely, Grade 0 (no signs of gingival enlargement); Grade I (enlargement confined to interdental papilla); Grade II (enlargement involving interdental papilla and the marginal gingiva); Grade III (enlargement covering three quarters or more of the crown) [10].

As a result of the bacterial plaque build-up that frequently occurs along with these enlargements over time, regular professional oral prophylaxis is required, as is excellent patient compliance. Patient education and compliance throughout dental treatment are the most important factors. Understanding the pathologic alterations underlying gingival enlargement is the first step in treating it [11]. Local methods can successfully treat growths brought on by inflammation alone, and meticulous dental hygiene helps to prevent recurrence.

In the current case, the patient reported having gingival enlargement in the anterior region of both upper and lower arches. Inflammation was reduced following phase I therapy. However, persistent enlargement necessitated surgical excision of the lesion. Gingivectomy showed excellent aesthetic results at the 15-day follow-up visit which was similar to the results obtained in a study by Lione et al. who observed satisfactory healing and post-operative results following gingivectomy using a scalpel [12]. Despite having the lesion treated, ongoing long-term monitoring is necessary to determine whether the lesion is regressing.

## Conclusions

Understanding the underlying pathologic alterations and the reason for gingival enlargement is the foundation for treating it. Since it affects both function and aesthetics, gingival enlargement is of utmost concern to the patient. The biggest benefit for the patient in cases of excessive enlargement will come from an appropriately scheduled surgical procedure to bring back the size of the tissue to a normal contour, lessening the number of required clinical visits and improving the individual's standard of life.
